# Supplementary figures and images for: Validity of Bioimpedance Equations to Evaluate Fat-Free Mass and Muscle Mass in Severely Malnourished Anorectic Patients
Source: J Clin Med. 2020 Nov 14;9(11):3664. doi: 10.3390/jcm9113664 (PMC7698304; doi:10.3390/jcm9113664)

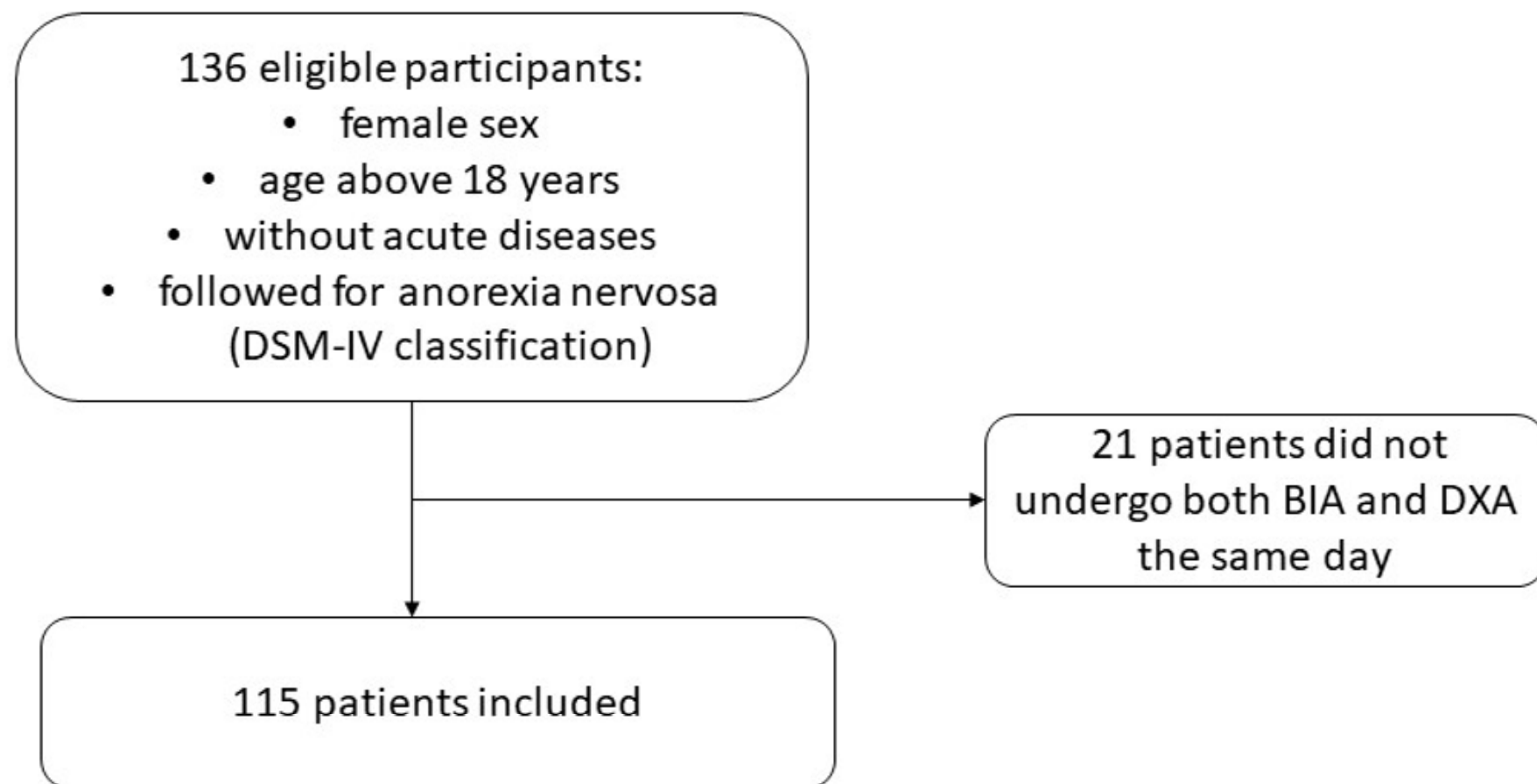

**Supplemental figure 1: Participants Flow Chart**

Supplement: Supplementary file 1 [file jcm-09-03664-s001.pdf]
